# High prevalence of carpal tunnel syndrome in individuals with rare nerve growth factor-beta mutation

**DOI:** 10.1093/braincomms/fcaa085

**Published:** 2020-06-25

**Authors:** Mikael Ridderström, Mats Svantesson, Oumie Thorell, Theofilos Magounakis, Jan Minde, Håkan Olausson, Saad S Nagi

**Affiliations:** f1 Department of Orthopaedics, Gällivare Hospital, Gällivare, Sweden; f2 Department of Clinical Neurophysiology, Linköping University, Linköping, Sweden; f3 Department of Biomedical and Clinical Sciences, Center for Social and Affective Neuroscience, Linköping University, Linköping, Sweden; f4 Department of Integrative Physiology, School of Medicine, Western Sydney University, Sydney, Australia; f5 Department of Radiology, Piteå Älvdals Hospital, Piteå, Sweden; f6 Division of Orthopaedics, Department of Surgery and Perioperative Science, Umeå University Hospital, Umeå, Sweden

**Keywords:** carpal tunnel syndrome, hereditary sensory and autonomic neuropathy type V, nerve conduction, nerve growth factor, ultrasonography

## Abstract

In Sweden, a large family with a point mutation in the nerve growth factor-beta gene has previously been identified. The carriers of this mutation have reduced small-fibre density and selective deficits in deep pain and temperature modalities. The clinical findings in this population are described as hereditary sensory and autonomic neuropathy type V. The purpose of the current study was to investigate the prevalence of carpal tunnel syndrome in hereditary sensory and autonomic neuropathy type V based on clinical examinations and electrophysiological measurements. Furthermore, the cross-sectional area of the median nerve at the carpal tunnel inlet was measured with ultrasonography. Out of 52 known individuals heterozygous for the nerve growth factor-beta mutation in Sweden, 23 participated in the current study (12 males, 11 females; mean age 55 years; range 25–86 years). All participants answered a health questionnaire and underwent clinical examination followed by median nerve conduction study in a case–control design, and measurement of the nerve cross-sectional area with ultrasonography. The diagnosis of carpal tunnel syndrome was made based on consensus criteria using patient history and nerve conduction study. The prevalence of carpal tunnel syndrome in the hereditary sensory and autonomic neuropathy group was 35% or 52% depending on whether those individuals who had classic symptoms of carpal tunnel syndrome but negative nerve conduction studies were included or not. Those who had a high likelihood of carpal tunnel syndrome based on classic/probable patient history with positive nerve conduction study had a significantly larger median nerve cross-sectional area than those who had an unlikely patient history with negative nerve conduction study. The prevalence of carpal tunnel syndrome was 10–25 times higher in individuals heterozygous for the nerve growth factor-beta mutation than the general Swedish population. Further studies are needed to better understand the underlying pathophysiological mechanisms.

## Graphical Abstract

**Graphical Abstract:**
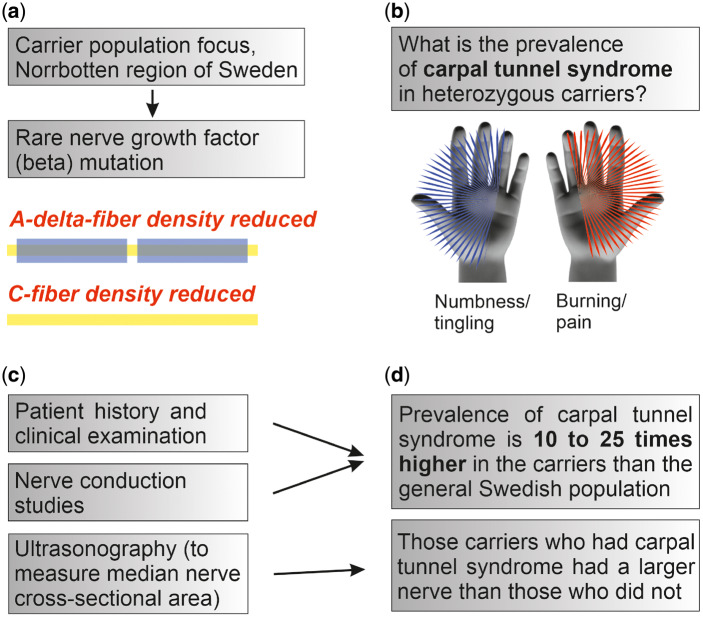

Abbreviated summary

In a unique group of heterozygous carriers of a rare mutation in the nerve growth factor-beta gene, we investigated the prevalence of carpal tunnel syndrome and found it to be manifold higher in the carriers than the general Swedish population.

## Introduction

Hereditary sensory and autonomic neuropathies are rare genetic conditions that can be classified into five subtypes (HSANs I–V) based on their mode of inheritance, natural history, pathology, biochemical and neurophysiological features and degree of autonomic involvement (Dyck *et al.*, 1983). We have previously identified a large family residing in northern Sweden with a condition best fitting the HSANV subtype (Minde *et al.*, 2004). They have a point mutation in the gene encoding nerve growth factor-beta on chromosome 1p11.2–p13.2, and the pattern of inheritance is autosomal recessive (Einarsdottir *et al.*, 2004).

Individuals with HSANV have a normal cognitive function (Minde *et al.*, 2004). Heterozygous carriers—the focus of the current study—have milder symptoms than their homozygous counterparts, a moderate reduction in Aδ and C fibres in sural nerve biopsies, and the clinical picture ranges from asymptomatic cases to Charcot arthropathy starting in adult life (20–30 years old in some cases) but is most common above 70 years (Minde *et al.*, 2009). In a previous study on the phenotype of heterozygous carriers, a serendipitous finding was that a proportion of them were vulnerable to carpal tunnel syndrome (CTS) (Minde *et al.*, 2009). However, that study was not designed to assess the prevalence of CTS, and the clinical examination was unstructured. Evidence from clinical studies suggests a disturbance in small-fibre function in patients with CTS, including reduced laser-evoked brain potentials (Arendt-Nielsen *et al.*, 1991), increased thermal detection thresholds (Schmid *et al.*, 2014), reduced intraepidermal nerve fibre density (Schmid *et al.*, 2014) and decreased sympathetic skin response (Kiylioglu *et al.*, 2005; cf. Bayrak *et al.*, 2007) and vasomotor function (Wilder-Smith *et al.*, 2003). The prevalence of CTS in the general population in Sweden is estimated to be 2.1–3.6% (Atroshi *et al.*, 1999).

CTS is a median nerve compression neuropathy at the wrist presenting as a varied combination of numbness, tingling and pain in the median nerve territory of the hand that sometimes radiates up the forearm. In mild-to-moderate cases, the symptoms are intermittent and often worse at night and after physical activity. Severe cases have persistent sensory loss, motor disturbances with weak thumb abduction and thenar muscle atrophy (Dawson, 1993). Diagnosis is classically made by a combination of patient history, clinical examination and nerve conduction study (NCS).

Ultrasonography has emerged as a possible adjunct to NCS in the diagnosis of CTS since it is less time consuming and not unpleasant for the patient (Ghasemi-Rad *et al.*, 2014). In CTS, the nerve becomes swollen at the carpal tunnel inlet and a large cross-sectional area (CSA) at this level is a diagnostic feature. Varied concordance with NCS has been shown good in some studies and poor in others. Many have tried to establish the best cut-off value for the median nerve CSA at the carpal tunnel inlet in the diagnosis of CTS, but the proposed values vary widely from 6.5 to 15 mm^2^ (Pinilla *et al.*, 2008; Sarraf *et al.*, 2014).

For epidemiological research, a consensus regarding the classification of CTS was presented by Rempel *et al.* (1998) where patient history was divided into three categories: classic/probable, possible and unlikely. This was combined with NCS being either negative or positive (see Tables 1 and 2).

**Table 1:** Classification of patient history for CTS

Symptoms	Description
Classic/probable	Numbness, tingling, burning or pain in at least two digits of digiti I, II and IIIWrist pain, palm pain or proximal radiation is allowed
Possible	Numbness, tingling, burning or pain in at least one digit of digiti I, II and III
Unlikely	No symptoms in digitus I, II or III

Reproduced from Rempel *et al.* (1998).

**Table 2:** Consensus criteria for CTS based on patient history and NCS

Patient history	NCS	Ordinal likelihood of CTS
Classic/probable	Positive	+++
Possible	Positive	++
Classic/probable	Negative	+/−^a^
Possible	Negative	−
Unlikely	Positive	−
Unlikely	Negative	−−

From Rempel *et al.* (1998).

a No consensus whether it should be classified as CTS or not.

The aim of the current study was to determine the prevalence of CTS in the heterozygous group, using the aforementioned consensus criteria (Rempel *et al.*, 1998) and to measure the CSA of the median nerve at the carpal tunnel inlet with ultrasonography.

## Materials and methods

### Study group

There are 52 known individuals who are heterozygous for the 1p11.2–p13.2 mutation in Sweden. Of these, four were excluded from the study due to age (under 15 or above 85 years), and another four were excluded because they had previously participated in at least one study and had expressed no interest in further studies. Out of 44 heterozygotes who were invited to participate in the study, 23 volunteered (44% of known heterozygotes), while 29 chose not to participate because of work, study, length of travel or personal reasons. There were 12 males (52%) and 11 females (48%) in the HSANV group. The mean age was 55 years (range 25–86) and the median age was 59 years. Five relatives without the nerve growth factor-beta mutation also volunteered to participate [1 male, 4 females; mean age 54 (range 25–72) years; median age, 59 years]; data for these are shown in Supplementary Table 1. To compare NCS data, a group of 30 healthy participants (20–79 years, 18 females, 12 males) served as controls. All volunteers gave informed written consent to participate in the study, which was approved by the ethics committee of the Umeå University and complied with the revised Declaration of Helsinki.

### Patient history and clinical examination

Each patient received by mail a screening questionnaire regarding their general health, medications, symptoms of neuropathy and whether their occupation included work with vibrating tools. Manual work with repetitive wrist motion, forceful hand movements and the use of vibrating tools increase the risk of developing CTS (Newington *et al.*, 2015; Guan *et al.*, 2018).

Clinical examination was performed by a single physician (M.R.) working as an orthopaedic surgeon. Patient history was collected and classified as classic/probable, possible or unlikely for CTS as described in Tables 1 and 2. If the patient previously had surgery for suspected CTS and was relieved of symptoms, this was classified as classic/probable CTS. The surgically treated wrists are described separately in the Results.

Clinically all wrists were assessed for Tinel’s sign (positive or negative) and Phalen’s test for 60 s (positive or negative and after how many seconds did the symptoms occur in each hand). Phalen’s test involves a prolonged maximal flexion of the patient’s wrist to see whether this triggers or aggravates paresthaesia in the median nerve territory, and Tinel’s sign involves tapping over the carpal tunnel to see whether this elicits paresthaesia/tingling (Ghasemi-Rad *et al.*, 2014).

It was also noted whether the patient had symptoms and clinical signs of ulnar entrapment based on patient history with numbness/tingling in an ulnar nerve distribution and/or weakness of ulnar nerve-innervated musculature. During the clinical examination, motor and sensory functions of the ulnar nerve were tested and Tinel’s test was performed over the nerve in the cubital tunnel as well as in Guyon’s canal. Patients were also asked if they had a history of neck pain and pain radiating down the arm to screen for cervical radiculopathy.

### Nerve conduction studies

NCS was performed by an experienced biomedical technician (O.T.), who was blind to patient history and results from the clinical examination, following a standardized procedure used in clinical practice at the Department of Clinical Neurophysiology at the Linköping University Hospital. The results were analysed by a clinical neurophysiologist (M.S.). Both motor and sensory studies were done on the median nerve with a comparative sensory study of the ulnar nerve. For motor measurements, stimulation was performed proximal to the wrist and registration was done over abductor pollicis brevis and abductor digiti minimi muscles for the median and ulnar nerves, respectively. Sensory responses were recorded orthodromic by digital stimulation with ring electrodes and registration proximal to the wrist over the corresponding nerves (third and fourth fingers for the median nerve and the fourth finger for the ulnar nerve; for the fourth finger, the same distance between stimulation and registration was used for both median and ulnar recordings). The electrophysiological diagnosis of CTS was based on a significant difference in sensory conduction velocity between the median and ulnar nerves, i.e. a difference in distal latency of >0.6 ms when stimulating at the fourth digit and recording compound sensory potentials from the median and ulnar nerves. In moderate-to-severe cases, there was also an increased distal motor latency for the median nerve (>4.2 ms) (Table 3). This is a modified version of the criteria proposed by Bland (2000) in which fewer grades are used, the threshold values are adapted to local experience and, instead of using the biphasic sensory response of the fourth finger, the latency difference between the median and ulnar nerves is used to define a very mild CTS.

**Table 3:** Quantitative classification of median nerve dysfunction during NCS

1. Very mild	Increased sensory latency median nerve digit IV >0.6 ms
2. Mild	1+ decreased sensory conduction velocity/amplitude digit III
3. Moderate	2+ increased distal motor latency in median nerve >4.2 ms
4. Severe	Same as 3 but with no sensory response

### Ultrasonography

All ultrasound examinations were performed by a single radiologist (T.M.) with 12 years’ experience of musculoskeletal ultrasound examinations. The radiologist was blinded to the results from the clinical examination and the nerve conduction studies. The examinations were performed using a General Electric Logic E9 ultrasound machine with an ML-6/15 linear array transducer (GE Healthcare, Chicago, IL, USA). All patients were seated during examination with the wrist supported on a gel pad, fingers extended, hand fully supinated, elbows flexed to 90° and shoulders in a neutral position. Transverse images of the median nerve were obtained at the carpal tunnel inlet at the level of the scaphoid bone. The CSA was measured perpendicular to the nerve using the trace volume function inside the hyperechoic rim of the nerve. Measurements were taken for both right and left wrists.

### Statistical analysis

A Chi-square test was used to assess differences between the HSANV and the control group regarding the occurrence of electrophysiological CTS. Kruskal–Wallis and *post hoc* Dunn’s multiple comparisons tests were used to compare ultrasonography results between patient groups based on the likelihood of CTS. We considered *P* < 0.05 as statistically significant. Data were analysed in SPSS (version 25; IBM Corporation, Armonk, NY, USA) and GraphPad Prism (version 6.07; GraphPad Software Inc., La Jolla, CA, USA).

### Data availability

Raw data were generated at the Department of Orthopaedics, Gällivare Hospital. Derived data supporting the findings of this study are available from the corresponding author on request.

## Results

### Clinical examination

Out of 23 patients who were tested bilaterally, 4 (17%) of these previously had surgery for CTS (3 bilaterally and 1 only on the left side) (Table 4). All were relieved of the symptoms they had before surgery and were therefore included in the classic/probable group. The patient who previously had surgery only on the left wrist had now developed classic/probable symptoms on the right wrist. With the inclusion of the surgically treated patients, there were 11 (48%) with a classic/probable history, 2 (9%) with a possible history and 10 (44%) with an unlikely history for CTS. All patients in the classic/probable and possible groups have (or previously had) bilateral symptoms.

**Table 4:** Clinical, electrophysiological and ultrasonography results of heterozygotes (Cases 1–23)

Case	Gender	Age	Risk factors	CTS surgery	History	Tinel’s R/L	Phalen’s R/L	NCS	Q R	Q L	CTSC	US R	US L
1	M	74			P	Neg/neg	Neg/neg	Pos	3	3	++	8	8
2	F	31			U	Neg/neg	Neg/30s	Neg	0	0	−−	9	9
3	F	52			C/P	Neg/neg	60s/20s	Neg	0	0	+/−	12	14
4	M	75			C/P	Pos/pos	10s/10s	Pos	4	4	+++	16	17
5	M	75	DM	Bilateral	C/P	Pos/neg	60s/30s	Pos	4	3	+++	8	10
6	F	75			P	Neg/neg	Neg/40s	Neg	3?^a^	0	−	9	8
7	F	57		Left	C/P	Neg/neg	Neg/40s	Pos	4	3	+++	13	11
8	M	25	VT		U	Neg/neg	Neg/neg	Neg	0	0	−−	7	8
9	M	58			C/P	Neg/neg	40s/40s	Neg	0	0	+/−	9	9
10	M	59	VT		U	Neg/neg	Neg/neg	Pos	4	4	−	12	13
11	F	26	Wrist frx		C/P	Neg/neg	40s/40s	Neg	0	0	+/−	5	6
12	M	26			U	Neg/neg	Neg/neg	Neg	0	0	−−	8	9
13	F	55			C/P	Neg/neg	30s/30s	Neg	0	0	+/−	8	8
14	M	86			U	Neg/neg	40s/40s	Pos	4	4	−	8	9
15	F	64	DM		U	Neg/neg	Neg/neg	Neg	0	0	−−	6	7
16	M	36	Scaphoid frx		C/P	Neg/neg	20s/20s	Pos	3	0	+++R, +/−L	8	11
17	F	61		Bilateral	C/P	Neg/neg	Neg/30s	Pos	2	2	+++	8	7
18	M	64			U	Neg/neg	Neg/neg	Neg	0	0	−−	6	6
19	F	82		Bilateral	C/P	Pos/neg	30s/30s	Pos	4	4	+++	7	8
20	M	59	VT		C/P	Neg/neg	45s/15s	Pos	3	4	+++	9	11
21	M	28			U	Neg/neg	Neg/neg	Neg	0	0	−−	6	9
22	F	70			U	Neg/neg	Neg/neg	Neg	0	0	−−	10	10
23	F	28			U	Neg/neg	Neg/neg	Neg	0	0	−−	5	5

C/P, classic/probable; CTSC, the likelihood of CTS based on the consensus criteria (Table 2); DM, diabetes mellitus; frx, fracture; L, left; P, possible; Q, quantity of changes seen on NCS (Table 3); R, right; U, unlikely; US, ultrasonography of median nerve CSA at carpal tunnel inlet (mm²); VT, work with vibrating tools.

a A marked decrease in motor amplitudes but close to a normal sensory response does not meet the sensory criteria. A median nerve dysfunction other than CTS was suspected.

In the 11 patients with classic/probable CTS, Phalen’s test was positive with a mean time of 32 s (range 10–60 s). Out of seven surgically treated wrists, six still had a positive Phalen’s test with a mean time of 37 s. In the two patients with a possible history of CTS, one had a negative Phalen’s test on both sides while the other had a negative test on the right side and a positive test after 40 s on the left side. For the 10 patients with an unlikely history of CTS, Phalen’s test was negative in eight and positive in two (one with a bilateral positive result after 40 s, and the other with a positive result after 30 s in the left hand only).

Out of the 46 examined wrists, Tinel’s sign was positive in 4 wrists (right side in 2 patients with previous bilateral surgery, and bilaterally in 1 patient with classic/probable history). Marked thenar atrophy was found in one patient with classic/probable history who had a positive Phalen’s test after 10 s bilaterally; the same patient also had symptoms and clinical features of ulnar entrapment in the elbow on the left side. Suspected ulnar entrapment in the elbow was also found in three more patients, two with mild ulnar symptoms bilaterally (one in the classic/probable group and the other in the unlikely group) and one with an affected left side (classic/probable group). Clinically there were no cases with suspected cervical radiculopathy.

The screening questionnaire showed that there were no cases with rheumatic disease or hypothyroidism. Body mass index (BMI) and smoking habits were not assessed. Seven patients had other risk factors for CTS, but none had more than one risk factor (Table 4). Two patients had type 2 diabetes mellitus (one in the unlikely group and the other in the classic/probable group who previously had surgery bilaterally). Two patients reported previous wrist surgeries: one for a wrist fracture and the other for a scaphoid fracture (both in classic/probable group). Three patients reported working with vibrating tools (one in the classic/probable group and two in the unlikely group).

### Nerve conduction studies

Out of 23 patients, NCS identified 10 (44%) patients fulfilling the electrophysiological criteria for CTS (9 bilateral and 1 in the right hand only even though the participant had bilateral symptoms). Out of these 19 wrists, the median nerve dysfunction was classified as severe in 11 (58%), moderate in 6 (32%) and mild in 2 (10%). Of these 10 patients, 7 were in the classic/probable group, 1 in the possible group and 2 in the unlikely group. All patients with a history of corrective surgery for CTS fulfilled the NCS diagnostic criteria. For age-matched analysis of NCS controls, 2 patients who were above 79 years were excluded, leaving 8 out of 21 (38%) patients with CTS. In the control group, 2 out of 30 subjects (7%) were classified as having CTS of a very mild degree. The Chi-square test showed a significant difference (Chi-square score 7.74; *P* = 0.005) between the groups. NCS data are summarized in Supplementary Table 2.

### Prevalence of carpal tunnel syndrome

The result of implementing the consensus criteria for CTS (Rempel *et al.*, 1998) is presented in Table 5. The prevalence of CTS in the HSANV group was 8 (35%) or 12 (52%) out of 23 depending on whether the 4 patients with classic/probable history but negative NCS (Cases 3, 9, 11 and 13 in Table 4) were included or not (Table 6). These four cases had, in common, symptoms that were mild and intermittent, yet clinically definitely classic/probable CTS and the physical examination with Phalen’s test was positive.

**Table 5:** Likelihood of CTS in the HSANV group based on the consensus criteria

Likelihood	Number of patients	Female	Male	Mean age (range)	Risk factors
+++	7 (30%)	3 (43%)	4 (57%)	64 (36–82)	3 (43%)
++	1 (4%)	0	1 (100%)	74	0
+/−	4 (17%)	3 (75%)	1 (25%)	48 (26–58)	1 (25%)
−	3 (13%)	1 (33%)	2 (67%)	73 (59–86)	1 (33%)
−−	8 (35%)	4 (50%)	4 (50%)	42 (25–70)	2 (25%)
Total	23	11	12	55 (25–86)	7

**Table 6:** Prevalence of CTS in the HSANV group

Likelihood	Number of patients	Female	Male
+++ or ++	8 out of 23 (35%)	3 (38%)	5 (63%)
+++, ++ or +/−	12 out of 23 (52%)	6 (50%)	6 (50%)

**Table 7:** CSA at carpal tunnel inlet for the median nerve

Likelihood of CTS	Nerve CSA	Nerve CSA (operated wrists excluded)
+++	10.2 ± 3.3 (7.0–17.0) *N* = 13	12.3 ± 3.7 (8.0–17.0) *N* = 6
++	8.0 ± 0.0 (8.0) *N* = 2	None in the group had surgery
+/−	9.1 ± 2.8 (5.0–14.0) *N* = 9	None in the group had surgery
−	9.8 ± 2.1 (8.0–13.0) *N* = 6	None in the group had surgery
−−	7.5 ± 1.7 (5.0–10.0) *N* = 16	None in the group had surgery

All values are presented in mm² as mean ± standard deviation (range).

*N*, number of wrists.

There were two patients with an unlikely history for CTS but with NCS showing severe median nerve dysfunction bilaterally (Cases 10 and 14 in Table 4). However, these did not meet the consensus criteria for CTS diagnosis. There was also one uncertain case (Case 6, Table 4) with possible history and a right-sided marked decrease in motor amplitudes of the median nerve on NCS but close to normal sensory response, thus not fulfilling the sensory criteria. A median nerve dysfunction other than CTS was suspected.

### Ultrasonography

Individual patient data for nerve CSA are shown in Table 4. When all patients were compared based on CTS likelihood, no statistically significant difference in nerve CSA was found (Kruskal–Wallis test: *P* = 0.1202). Notably, all patients with surgically treated wrists had normal or close to normal CSA (mean 8.4 mm^2^) despite positive NCS and they were all in the +++ group (Table 4). Therefore, we sub-analysed the results with the surgically treated wrists excluded (Table 7), and this revealed a statistically significant difference in nerve CSA between the groups (Kruskal–Wallis test: *P* = 0.0332). *Post hoc* analysis revealed a significant difference in CSA between the classic/probable (+++) and unlikely (−) groups [mean ± standard deviation; (+++), 12.3 ± 3.7 mm^2^; (−), 7.5 ± 1.7 mm^2^; Dunn’s multiple comparisons test: *P* = 0.0296]. No difference was found for the other patient groups (for nerve CSA of patients’ relatives without the mutation see Supplementary Table 1).

## Discussion

We found that individuals heterozygous for a point mutation in the nerve growth factor-beta gene resulting in HSANV have a very high prevalence of CTS. There was good-to-excellent concordance between patient history, Phalen’s test and NCS results. Tinel’s sign was not a reliable clinical test for the study group. The data analysis was complicated by the fact that the criteria used (Rempel *et al.*, 1998) did not define a conclusive classification for the +/− group. Depending on whether these were classified as CTS or not, the prevalence of CTS was 10–25 times higher (35–52%) for the study group than the general Swedish population (2.1–3.6%) (Atroshi *et al.*, 1999). This finding is consistent with neurophysiological, psychophysical and histological evidence from clinical studies that the small-fibre function is affected in entrapment neuropathies (Arendt-Nielsen *et al.*, 1991; Schmid *et al.*, 2014).

When the surgically treated wrists were included, we found no statistically significant difference in the median nerve CSA at the carpal tunnel inlet for those with CTS in the HSANV group compared to those without CTS. However, when the surgically treated wrists were excluded, we found a significant difference in the median nerve CSA at the carpal tunnel inlet between the patients who had a high likelihood of CTS based on classic/probable patient history with positive NCS compared to those who had an unlikely patient history with negative NCS. Previous reports on the use of ultrasound in other clinical populations have also shown an increase in CSA of the median nerve in CTS (Pinilla *et al.*, 2008; Sarraf *et al.*, 2014). It remains to be tested whether the same applies to homozygous carriers who have a more severe reduction in small fibres than in heterozygous carriers; however, none of the three known cases of the homozygous phenotype have CTS (Einarsdottir *et al.*, 2004). The mechanisms underpinning nerve enlargement in response to compression are not entirely clear but based on nerve biopsies likely involve oedema, thickening of microvascular walls and fibrosis at the injury site (reviewed in Rempel *et al.*, 1999).

The current study population did not have abundant risk factors for CTS, but a limitation of the current study is that we did not assess the BMI and smoking habits. However, none of the participants were visibly obese and it seems unlikely that BMI, or smoking habits for that matter, could explain the markedly high prevalence of CTS. Indeed, the reason for a very high prevalence of CTS in heterozygotes for the nerve growth factor-beta mutation remains unknown. Diabetic polyneuropathy is associated with an increased risk of CTS (Moon *et al.*, 2020) with several metabolic and vascular factors involved that make the hyperglycaemic nerve more susceptible to compression at the carpal tunnel (e.g. Suzuki *et al.*, 1994). CTS is also commonly reported in chemotherapy-induced neuropathy (Miaskowski *et al.*, 2017).

A recent mouse model of HSANV heterozygotes displayed reduced nociception and skin innervation but no learning or memory deficits thus excluding haploinsufficiency as a mechanistic explanation (Testa *et al.*, 2019). A significant reduction in the nerve CSA was found in the HSANV mouse model; the number of myelinated axons was unaffected, but the unmyelinated axons were significantly reduced (Testa *et al.*, 2019). In our patient population, a comparison between CTS positive (+++) and CTS negative (−) patients revealed larger nerve CSA in the CTS condition. The CSA of HSANV patients without CTS was similar to relatives of these patients without the mutation (data in Supplementary Table 1) and the published normative data (e.g. Koyuncuoglu *et al.*, 2005).

When comparing the sensory values of the ulnar nerve of the fourth finger between the HSANV and the control group in a *post hoc* analysis, there were significant differences in both amplitude and conduction velocity (data in Supplementary Table 2). Since the ulnar nerve does not pass through the carpal tunnel, CTS cannot explain this, and it may reflect a more general nerve vulnerability in the HSANV group. Here, the ulnar recordings were not performed over the elbow, so cubital tunnel syndrome cannot be ruled out—the second most common compression neuropathy after CTS in the upper extremity (Seror and Nathan, 1993). Whether the high prevalence of CTS in HSANV patients is due to focal or polyneuropathy cannot be confirmed from current observations and requires further investigations including conduction studies involving multiple nerves and body sites.

It was previously reported that tactile directional sensibility, a sensitive measure of large (A-beta) fibre function, is normal in HSANV patients (Morrison *et al.*, 2011). In the current study, psychophysical testing according to the protocol defined in the Michigan Neuropathy Screening Instrument (Herman *et al.*, 2012) revealed that most patients were able to detect mechanical stimuli on the great toe, albeit to a variable degree of sensitivity, tested bilaterally using a 128-Hz tuning fork or a 100-mN monofilament (data in Supplementary Table 3). Several of these had foot deformities including Charcot joints. In a recent study, we screened for thermal function and found two patients in this cohort who had no cold (<20°C) or heat pain (>50°C) perception and yet their mechanical pain perception was intact, a feature that from convergent evidence is likely signalled by the large-diameter (A-beta) nociceptors (Nagi *et al.*, 2019).

It might be speculated that due to relative pain insensitivity, the HSANV patients use their wrists with excessive repetitive motion, force, impulsiveness or in extreme positions leading to repeated microtrauma, the potential role of which has been previously described in osteoarthritis (Radin *et al.*, 1991; Radin, 1995) and is consistent with a high prevalence of osteoarthritis and Charcot arthropathies in this group (Minde *et al.*, 2009). However, further studies on the pathophysiological mechanisms of CTS development in HSANV are needed.

## Supplementary Material

fcaa085_Supplementary_Data

## Abbreviations

CSA =: cross-sectional area
CTS =: carpal tunnel syndrome
HSAN =: hereditary sensory and autonomic neuropathy
NCS =: nerve conduction study
