# Supplementary material for: High prevalence of carpal tunnel syndrome in individuals with rare nerve growth factor-beta mutation
Source: Brain Commun. 2020 Jun 25;2(2):fcaa085. doi: 10.1093/braincomms/fcaa085 (PMC7472894; doi:10.1093/braincomms/fcaa085)
Supplement: fcaa085_Supplementary_Data [file fcaa085_supplementary_data.docx]

| **Supplementary Table 1** Clinical, electrophysiological and ultrasonography results of relatives (without *NGFB* mutation) of HSANV patients (cases 24–28) | | | | | | | | | | | | | |
| --- | --- | --- | --- | --- | --- | --- | --- | --- | --- | --- | --- | --- | --- |
| **Case** | **Gender** | **Age** | **Risk factors** | **CTS surgery** | **History** | **Tinel's R/L** | **Phalen's R/L** | **NCS** | **Q R** | **Q L** | **CTSC** | **US R** | **US L** |
| 24 | F | 72 | - |  | U | neg/neg | neg/neg | Neg | 0 | 0 | -- | 8 | 9 |
| 25 | F | 52 | - |  | U | neg/pos | 45s/30s | Neg | 0 | 0 | -- | 7 | 6 |
| 26 | M | 25 | VT |  | U | neg/neg | neg/neg | Neg | 0 | 0 | -- | 7 | 9 |
| 27 | F | 62 | - | Left | **C/P** | neg/neg | 15s/neg | **Pos** | 0 | **3** | **+/-R, +++L** | 8 | 8 |
| 28 | F | 59 | - |  | U | neg/neg | neg/neg | Neg | 0 | 0 | -- | 6 | 9 |
| VT, work with vibrating tools; C/P, classic/probable; U, unlikely; Q, quantity of changes seen on NCS (Table 3); CTSC, the likelihood of CTS based on the consensus criteria (Table 2); US, ultrasonography of median nerve cross-sectional area at carpal tunnel inlet (mm²); R, Right; L, Left. The mean ± standard deviation of nerve cross-sectional area for relatives with one surgically treated wrist removed is 7.7 ± 1.2 mm². | | | | | | | | | | | | | |

| **Supplementary Table 2** Age-matched analysis of nerve conduction studies for HSANV patients (n=8) and healthy participants (n=30) | | | | | | | | | |
| --- | --- | --- | --- | --- | --- | --- | --- | --- | --- |
|  | | | | Control Median (Min-Max) | | HSANV Median  (Min-Max) | | Significance (P < 0.05) | |
|  |  |  |  | Right | Left | Right | Left | Right | Left |
| Median nerve | Motor | | Distal latency (ms) | 3.23 (2.76-4.43) | 3.13 (2.71-4.17) | 4.06  (3.13-7.66) | 3.65 (3.07-6.72) | 0.000 | 0.000 |
|  |  |  | Amplitude (mV) | 10.45 (6.10-15.60) | 10.05 (4.70-14.10) | 6.10 (1.00-12.40) | 6.80 (2.50-12) | 0.000 | 0.002 |
|  |  |  | Conduction velocity (m/s) | 57.75 (50.20-91.30) | 58.00 (51.40-64.90) | 54.9* (38.90-62.30) | 57.30* (45.10-67.10) | 0.227 | 0.215 |
|  | Sensory | Digit III | Amplitude (µV) | 14.55 (5.50-38.60) | 13.60 (4.80-36.30) | 8.55* (1.70-30.20) | 10.10* (2.30-25) | 0.011 | 0.114 |
|  |  |  | Conduction velocity (m/s) | 54.70 (44.40-65.70) | 56.95 (44.40-65.70) | 46.55* (28.10-58.00) | 49.00* (33.30-60.3) | 0.000 | 0.002 |
|  |  | Digit IV | Amplitude (µV) | 7.70 (1.10-19.50) | 7.25 (2.40-20.50) | 3.95* (0.94-23.10) | 4.9* (1.40-24.40) | 0.027 | 0.182 |
|  |  |  | Conduction velocity (m/s) | 54.90 (39.70-64.90) | 55.65 (41.10-64.00) | 44.15* (35.70-58.10) | 47.00* (33.70-59.10) | 0.000 | 0.005 |
|  |  |  | Latency difference (ms) | -0.0500 (-0.26-0.94) | 0.0000 (-0.21-0.78) | 0.1050 (-0.21-0.79) | 0.2100 (-0.26-0.57) | 0.005 | 0.160 |
| Ulnar nerve |  |  | Amplitude (µV) | 7.50 (1.80-16.20) | 5.80 (1.40-13.90) | 3.35* (1.10-12.30) | 3.35* (1.50-14.60) | 0.000 | 0.033 |
|  |  |  | Conduction velocity (m/s) | 54.60 (43.30-63.10) | 57.15 (49.00-63.10) | 49.55* (42.90-56.60) | 52.65* (45.40-66.70) | 0.005 | 0.008 |

 * Four patients (right) and six patients (left) with no response. P < 0.05 shown in red.

| **Supplementary Table 3** Results of the Michigan Neuropathy Screening Instrument in HSANV patients (cases 1–23) and relatives (without *NGFB* mutation) of HSANV patients (cases 24–28) | | | | | | | | | | | | | |
| --- | --- | --- | --- | --- | --- | --- | --- | --- | --- | --- | --- | --- | --- |
| **Case** | **Appearance of foot R** | **Ulceration R** | **Ankle reflex R** | **Vibration  128 Hz R** | **Monofilament 100 mN R** | **Appearance of foot L** | **Ulceration L** | **Ankle reflex L** | **Vibration  128 Hz L** | **Monofilament 100 mN L** | **Sum clinical tests (0–10)** | **Self-reported screening (0–13)** | **Total score (clinical and screening)** |
| **1** | 0 | 0 | 1 | 1 | 0.5 | 0 | 0 | 1 | 1 | 0 | 4.5 | 2 | 6.5 |
| **2** | 0 | 0 | 0 | 0 | 0.5 | 0 | 0 | 0 | 0 | 0.5 | 1 | 1 | 2 |
| **3** | 0 | 0 | 0 | 0.5 | 0 | 0 | 0 | 0 | 0.5 | 0 | 1 | 1 | 2 |
| **4** | 1 | 0 | 1 | 1 | 1 | 1 | 0 | 1 | 1 | 1 | 8 | 5 | 13 |
| **5** | 0 | 0 | 0 | 0.5 | 0.5 | 0 | 0 | 1 | 0.5 | 0.5 | 3 | 0 | 3 |
| **6** | 1 | 0 | 1 | 0.5 | 0 | 1 | 0 | 1 | 0.5 | 0 | 5 | 2 | 7 |
| **7** | 1 | 0 | 0 | 0.5 | 0 | 0 | 0 | 0 | 0.5 | 0 | 2 | 3 | 5 |
| **8** | 0 | 0 | 0 | 0 | 0 | 0 | 0 | 0 | 0 | 0 | 0 | 5 | 5 |
| **9** | 0 | 0 | 0 | 0 | 0 | 0 | 0 | 0 | 0 | 0 | 0 | 0 | 0 |
| **10** | 1 | 0 | 0 | 0 | 0 | 0 | 0 | 0 | 0 | 0 | 1 | 1 | 2 |
| **11** | 0 | 0 | 0 | 0 | 0 | 0 | 0 | 0 | 0 | 0 | 0 | 3 | 3 |
| **12** | 0 | 0 | 0 | 0 | 0 | 0 | 0 | 0 | 0 | 0 | 0 | 1 | 1 |
| **13** | 0 | 0 | 0 | 0.5 | 0 | 0 | 0 | 0 | 0.5 | 0.5 | 1.5 | 5 | 6.5 |
| **14** | 1 | 0 | 1 | 0.5 | 1 | 1 | 0 | 1 | 0.5 | 1 | 7 | 2 | 9 |
| **15** | 1 | 0 | 0 | 0 | 0 | 1 | 0 | 0 | 0 | 0 | 2 | 2 | 4 |
| **16** | 1 | 0 | 0 | 0 | 0 | 1 | 0 | 0 | 0 | 0 | 2 | 6 | 8 |
| **17** | 1 | 0 | 1 | 0.5 | 0 | 1 | 0 | 0 | 0.5 | 0 | 4 | 0 | 4 |
| **18** | 0 | 0 | 0 | 0 | 0 | 0 | 0 | 0 | 0 | 0 | 0 | 0 | 0 |
| **19** | 1 | 0 | 0 | 1 | 1 | 1 | 0 | 0 | 1 | 1 | 6 | 3 | 9 |
| **20** | 0 | 0 | 0 | 0.5 | 0 | 0 | 0 | 0 | 0.5 | 0 | 1 | 1 | 2 |
| **21** | 0 | 0 | 0 | 0 | 0 | 0 | 0 | 0 | 0 | 0 | 0 | 0 | 0 |
| **22** | 0 | 0 | 0 | 0 | 0 | 0 | 0 | 0 | 0 | 0 | 0 | 2 | 2 |
| **23** | 0 | 0 | 0 | 0 | 0 | 0 | 0 | 0 | 0 | 0 | 0 | 0 | 0 |
|  | | | | | | | | | | | | | |
| **24** | 1 | 0 | 0 | 1 | 0.5 | 1 | 0 | 1 | 0 | 0.5 | 5 | 2 | 7 |
| **25** | 0 | 0 | 0 | 0 | 0 | 0 | 0 | 0 | 0 | 0 | 0 | 3 | 3 |
| **26** | 0 | 0 | 0 | 0 | 0 | 0 | 0 | 0 | 0 | 0 | 0 | 0 | 0 |
| **27** | 0 | 0 | 0 | 0.5 | 0.5 | 1 | 0 | 0 | 0 | 0.5 | 2.5 | 6 | 8.5 |
| **28** | 1 | 0 | 0 | 0.5 | 1 | 0 | 0 | 0 | 0.5 | 0.5 | 3.5 | 1 | 4.5 |

Appearance of foot normal? If yes, marked as 0. If no, marked as 1 (deformities). Ulceration: 0 = Absent; 1 = Present. Ankle reflex: 0 = Present; 0.5 = Present with reinforcement (Jendrassic maneuver); 1 = Absent. Vibration perception (great toe): 0 = Present; 0.5 = Decreased; 1 = Absent. Monofilament detection (great toe): 0 = Normal; 0.5 = Reduced; 1 = Absent. R, Right. L, Left. The testing was performed according to the published criteria (e.g. Herman *et al.*, 2012).
